# Single-molecule optical mapping of the distribution of DNA phosphorothioate epigenetics

**DOI:** 10.1093/nar/gkab169

**Published:** 2021-03-25

**Authors:** Yue Wei, Qinqin Huang, Xihao Tian, Mingmin Zhang, Junkai He, Xingxiang Chen, Chao Chen, Zixin Deng, Zhiqiang Li, Shi Chen, Lianrong Wang

**Affiliations:** Ministry of Education Key Laboratory of Combinatorial Biosynthesis and Drug Discovery, School of Pharmaceutical Sciences, Wuhan University, Wuhan 430071, China; Taihe Hospital, Hubei University of Medicine, Shiyan 442000, Hubei, China; Department of Burn and Plastic Surgery, Division of Wound Repair, Shenzhen Institute of Translational Medicine, The First Affiliated Hospital of Shenzhen University, Shenzhen Second People's Hospital, Shenzhen 518035, China; Ministry of Education Key Laboratory of Combinatorial Biosynthesis and Drug Discovery, School of Pharmaceutical Sciences, Wuhan University, Wuhan 430071, China; Department of Molecular Pathology, The Second Affiliated Hospital, Academy of Medical Sciences of Zhengzhou University, Zhengzhou 450000, China; Ministry of Education Key Laboratory of Combinatorial Biosynthesis and Drug Discovery, School of Pharmaceutical Sciences, Wuhan University, Wuhan 430071, China; Ministry of Education Key Laboratory of Combinatorial Biosynthesis and Drug Discovery, School of Pharmaceutical Sciences, Wuhan University, Wuhan 430071, China; Ministry of Education Key Laboratory of Combinatorial Biosynthesis and Drug Discovery, School of Pharmaceutical Sciences, Wuhan University, Wuhan 430071, China; Ministry of Education Key Laboratory of Combinatorial Biosynthesis and Drug Discovery, School of Pharmaceutical Sciences, Wuhan University, Wuhan 430071, China; Department of Neurosurgery, Zhongnan Hospital, Wuhan University, Wuhan 430071, Hubei, China; Ministry of Education Key Laboratory of Combinatorial Biosynthesis and Drug Discovery, School of Pharmaceutical Sciences, Wuhan University, Wuhan 430071, China; Department of Neurosurgery, Zhongnan Hospital, Wuhan University, Wuhan 430071, Hubei, China; Ministry of Education Key Laboratory of Combinatorial Biosynthesis and Drug Discovery, School of Pharmaceutical Sciences, Wuhan University, Wuhan 430071, China; Department of Burn and Plastic Surgery, Division of Wound Repair, Shenzhen Institute of Translational Medicine, The First Affiliated Hospital of Shenzhen University, Shenzhen Second People's Hospital, Shenzhen 518035, China; Ministry of Education Key Laboratory of Combinatorial Biosynthesis and Drug Discovery, School of Pharmaceutical Sciences, Wuhan University, Wuhan 430071, China; Department of Burn and Plastic Surgery, Division of Wound Repair, Shenzhen Institute of Translational Medicine, The First Affiliated Hospital of Shenzhen University, Shenzhen Second People's Hospital, Shenzhen 518035, China; Department of Neurosurgery, Zhongnan Hospital, Wuhan University, Wuhan 430071, Hubei, China

## Abstract

DNA phosphorothioate (PT) modifications, with the nonbridging phosphate oxygen replaced by sulfur, governed by DndABCDE or SspABCD, are widely distributed in prokaryotes and have a highly unusual feature of occupying only a small portion of available consensus sequences in a genome. Despite the presence of plentiful non-PT-protected consensuses, DNA PT modification is still employed as a recognition tag by the restriction cognate, for example, DndFGH or SspE, to discriminate and destroy PT-lacking foreign DNA. This raises a fundamental question about how PT modifications are distributed along DNA molecules to keep the restriction components in check. Here, we present two single-molecule strategies that take advantage of the nucleophilicity of PT in combination with fluorescent markers for optical mapping of both single- and double-stranded PT modifications across individual DNA molecules. Surprisingly, PT profiles vary markedly from molecule to molecule, with different PT locations and spacing distances between PT pairs, even in the presence of DndFGH or SspE. The results revealed unprecedented PT modification features previously obscured by ensemble averaging, providing novel insights into the riddles regarding unusual target selection by PT modification and restriction components.

## INTRODUCTION

DNA phosphorothioate (PT) modification, in which the nonbridging oxygen in the sugar-phosphate backbone is replaced by sulfur, was initially recognized as a chemically synthesized phosphate analog and has been extensively utilized in oligonucleotide therapeutics (1). Surprisingly, it has been discovered that DNA PT modification occurs biologically in a sequence-selective and *R*_P_ configuration-specific manner in a wide range of bacteria and archaea expressing DndABCDE or SspABCD machinery (2–5). Nuclease resistance and redox and nucleophilic properties render PT modification a versatile player involved in multiple cellular processes (6–8). DNA PT modification serves as a constituent of prokaryotic defense systems, in which PT is used as a recognition tag by the restriction counterparts DndFGH, PbeABCD or SspE to discriminate self from non-self DNA and attack non-PT-modified invading DNA, similar to the role of DNA methylation in canonical restriction-modification (R-M) barriers (2,3,9,10). In some bacterial and archaeal strains, *dnd* and *ssp* systems exhibit the forms *dndABCDE* and *sspABCD*, respectively, devoid of restriction cognates; these forms are referred to as solitary PT modifications and endow cells with additional functions, including roles in the maintenance of the cellular redox state and epigenetic control of gene transcription (6).

DndABCDE-mediated DNA PT modifications occur in a variety of 4-bp consensus sequences, for example, 5′-G_PS_AAC-3′/5′-G_PS_TTC-3′ in *Escherichia coli* B7A and *Salmonella enterica* serovar Cerro 87 and 5′-G_PS_GCC-3′/5′-G_PS_GCC-3′ in *Pseudomonas fluorescens* pf0-1 (PS: phosphate-sulfur linkage) (6,11). In contrast to these bistranded PTs, 5′-C_PS_CA-3′ in *sspABCD*-expressing *Vibrio cyclitrophicus* FF75 is a single-stranded PT modification lacking PT in the complemented 5′-TGG-3′ (11). Notably, genomic PTs exhibit unusual features: (i) only 10–15% of the genome-wide 5′-GAAC-3′/5′-GTTC-3′ in B7A and Cerro 87 and 5′-CCA-3′ in FF75 are detected as PT modified, and (ii) PT modification does not occur consistently at a given consensus sequence in a cell population even in the presence of the active restriction counterpart, DndFGH or SspE (11,12). This feature distinguishes PT systems from classic methylation-based epigenetic and R-M systems. Given the evidence for incomplete PT modification of consensus sequences in a genome, questions arose about how Dnd and Ssp proteins select their targets in individual DNA molecules.

Three different approaches are currently applied to detect DNA PT epigenetics. Upon enzymatic digestion of DNA molecules, nuclease-resistant PT-linked dinucleotides, for example, d(G_PS_A), d(G_PS_T) and d(G_PS_G), are generated in addition to canonical monodeoxynucleotides, which allow the identification of PT modification by liquid chromatography-coupled tandem quadrupole mass spectrometry (LC-MS/MS) (13). The other two techniques, including single molecule real-time (SMRT) sequencing and deep sequencing of iodine-induced cleavage at PT (ICDS), do not involve enzymatic DNA hydrolysis and are utilized for genomic PT mapping (11,14). The oxygen-sulfur swap can alter the DNA polymerase kinetics used in SMRT sequencing, a sequencing-by-synthesis technology, making PT detectable on a genome-wide scale (11). However, this method suffers from low sensitivity owing to the need to distinguish PT-specific signals from noise. Alternatively, the ICDS method exploits the selective sensitivity of PT to induce DNA strand cleavage at PT sites, followed by ligation of a sequencing linker at the new 3′ end arising from iodine cleavage and then by Illumina sequencing (14). However, the two PT mapping methods are labor intensive and report on the population-averaged occurrence of PT. There is thus an urgent need to determine the distribution of PTs across single DNA molecules, which would reveal unprecedented PT modification behaviors previously obscured by ensemble averaging.

Here, we present two single-molecule strategies that take advantage of the nucleophilicity of PT in combination with fluorescent markers for tracking of PT modifications across single DNA molecules. We started with converting PT-modified 5′-C_PS_CA-3′/5′-TGG-3′ to single-stranded DNA nicks by iodine treatment, rendering them available for DNA polymerase-mediated incorporation of fluorescent nucleotides. Owing to DNA fragmentation resulting from iodine cleavage at bistranded PT sites, for example, 5′-G_PS_AAC-3′/5′-G_PS_TTC-3′, we developed a complementary method to detect PT modifications by selective chemical labeling of PTs in aqueous solution under mild conditions and using streptavidin-conjugated quantum dots (QDs) for visualization. When labeled DNA is stretched to linear form on glass slides, PT sites are visualized as fluorescent labels along the DNA backbone. The two optical mapping approaches enable us to discern PT profiles at the single-molecule level, which highlights the significant molecule-to-molecule PT heterogeneity and provides novel insights into the unusual target selection mechanism of Dnd and Ssp systems.

## MATERIALS AND METHODS

### Bacterial strains, plasmids and bacteriophages

All bacterial strains, plasmids and bacteriophages used in this study are listed in Supplementary Table S1. *Escherichia coli* strains were cultured at 37°C in Luria–Bertani (LB) broth or on agar plates. Phage plaque assays were performed as previously described by Xiong *et al.* (2).

### DNA preparation

The genomic DNA of *E. coli* 3234/A was extracted using a Genomic-tip 500/G (Qiagen) Kit when the OD_600_ reached 0.8. Plasmid DNA was isolated from the overnight culture of *E. coli* DH10B (pWHU3930) using the Plasmid DNA Mini Kit (Omega). NdeI-linearized pWHU3930 DNA was purified by a gel extraction kit (Omega) after agarose gel electrophoresis. λ genomes were prepared using a Lambda phage Genomic DNA Kit (Zoman Biotechnology).

Construction of the 15-kb-C_PS_CA DNA fragment was performed as follows: using λ DNA as template and 15-kb-U and 15-kb-C_PS_CA-D as primers, a 15-kb PCR product harboring a 5′-C_PS_CA-3′ site close to one end was amplified. In contrast, 15-kb-U and 15-kb-D were used as the primer pair to generate the 15-kb-CCA product, which has the same DNA sequence as 15-kb-C_PS_CA but lacks the PT modification.

Construction of the 15-kb-G_PS_AAC DNA fragment was performed as follows: using λ DNA as template and 15-kb-F and 15-kb-G_PS_AAC-R as primers, a 15-kb PCR product harboring a 5′-G_PS_AAC-3′ site close to one extremity was amplified. In contrast, 15-kb-F and 15-kb-R were used as the primer pair to generate the 15-kb-GAAC product, which has the same DNA sequence as 15-kb-G_PS_AAC but lacks the PT modification.

PCR products were gel purified using a gel extraction kit (Omega) after agarose gel electrophoresis. Primers are listed in Supplementary Table S2.

### Nick labeling of single-stranded PT modifications

Prior to iodine-mediated DNA nicking, pre-existing nicks or damage sites were blocked as described previously (14). To convert single-stranded PTs to DNA nicks, 1 μg of resultant DNA was treated with iodine by adding 5 μl of dibasic sodium phosphate buffer (500 mM, pH 9.0) and 5 μl of iodine solution (0.1 N, Coolaber) in a total volume of 50 μl. After incubation at 65°C for 5 min and cooling to 4°C, the reaction mixture was subjected to centrifugal ultrafiltration using Omega Membrane 10K (Pall) to remove iodine and salts. The purified DNA products were next mixed with 1 μl of shrimp alkaline phosphatase (New England Biolabs) and 5 μl of NEB CutSmart buffer to remove 3′-phosphates arising from iodine cleavage. After incubation at 37°C, the reaction was terminated by heating at 75°C for an additional 10 min. To fill PT-converted nicks, DNA samples were incubated with 1 μl of Taq DNA polymerase (New England Biolabs), 100 nM ddATP, 100 nM ddTTP, 100 nM ddGTP, 100 nM 5-propargylamino-dCTP-Cy5 (Jena Bioscience) and 5 μl of ThermoPol buffer (20 mM Tris-HCl (pH 8.8), 10 mM (NH_4_)_2_SO_4_, 10 mM KCl, 2 mM MgSO_4_ and 0.1% Triton® X-100) in a total volume of 50 μl. The mixture was incubated at 72°C for 2 h and then concentrated using Omega Membrane 10K to obtain a final volume of 20 μl. The labeled DNA was ready for molecular combing and imaging.

### Covalent labeling of PT modifications with IPB

The labeling reaction was carried out at 50°C for 12 h in the dark in 10 mM phosphate buffer (pH 7.0) with 20 μM 21mer-1PT or 2 μg of natural DNA and 20 mM IPB (Thermo Scientific) in a total volume of 10 μl. The excess IPB was removed by centrifugal ultrafiltration using Omega Membrane 1K (Pall). HPLC analysis and purification were performed using a Thermo Scientific UltiMate™ 3000 system. Aliquots of the oligonucleotide samples were loaded onto an Ultimate XB-C18 column (2.1 × 150 mm, 3 μm, Welch Materials Inc). The column was equilibrated with 95% buffer A (H_2_O, 10 mM ammonium acetate) and 5% buffer B (CH_3_CN). Elution was carried out by a three-step gradient program at a flow rate of 0.2 ml/min: 0–10 min, linear gradient to 10% buffer B; 10–20 min, constant 10% buffer B; 20–25 min, linear gradient to 97% buffer B. To determine the labeling yield by HPLC, 2 μM 20-mer PT-lacking oligonucleotide, 5′-GGAGCTGAGTGATCGCGTCA-3′, was used as an internal standard.

In terms of the covalent labeling of PT-containing DNA samples, 5 μg of genomic or plasmid DNA was incubated with 4 mM IPB at 50°C for 12 h in 10 mM phosphate buffer (pH 7.0). After removal of the excess IPB, the labeled DNA was ready for QD attachment. One hundred nanograms of biotinylated DNA was incubated with 50 nM Qdots Streptavidin Conjugate (QS605, Wuhan Jiayuan Quantum Dots) and 10 mM phosphate buffer (pH 7.0) in a total volume of 20 μl at room temperature for 60 min, followed by the addition of 250 nM YOYO-1 for an additional 30 min. A 0.5-μl reaction aliquot was mixed with 4.5 μl of phosphate buffer (0.1 M, pH 5.5) in preparation for molecular combing and imaging.

### DNA extension and optical imaging

Surfaces for DNA extension were prepared according to Michaeli *et al.* with minor modifications (22). Briefly, the coverslips and glass slides were incubated in a freshly made 1:2 (v/v) mixture of hydrochloric acid and nitric acid for 12 h in a chemical fume hood. After incubation, the coverslips and slides were washed extensively using ultrapure water and ethanol and air dried. Dry coverslips were immersed in a freshly made silane solution consisting of 12.4 μl of N-trimethoxysilylpropyl-N,N,N-trimethylammonium chloride (Alfa Aesar) and 6 μl of vinyltrimethoxysilane (Aladdin) in 50 ml of ultrapure water and incubated overnight at 65°C. The coverslips were thoroughly washed with ultrapure water five times and ethanol two times.

DNA molecules were next extended by pipetting a 5-μl droplet containing approximately 2.5 ng of DNA in 100 mM phosphate buffer (pH 5.5) between the microscope slide and silane-coated glass coverslips. The droplet was sucked between the glass surfaces by capillary force, leading to flow-induced stretching of the DNA molecules (28). The imaging of DNA samples was performed using a Nikon A1 confocal microscope with a 100× oil immersion objective. YOYO-1 iodide was excited using a 488-nm laser, whereas QDs and Cy3 were excited using a 561-nm laser. For 5-propargylamino-dCTP-Cy5, a 640-nm laser was used for excitation. All images were processed using ImageJ. The length of the DNA and distance between two fluorescent signals were measured manually using ImageJ.

## RESULTS

### Lighting up of single-stranded PT modification

**Figure 1.**
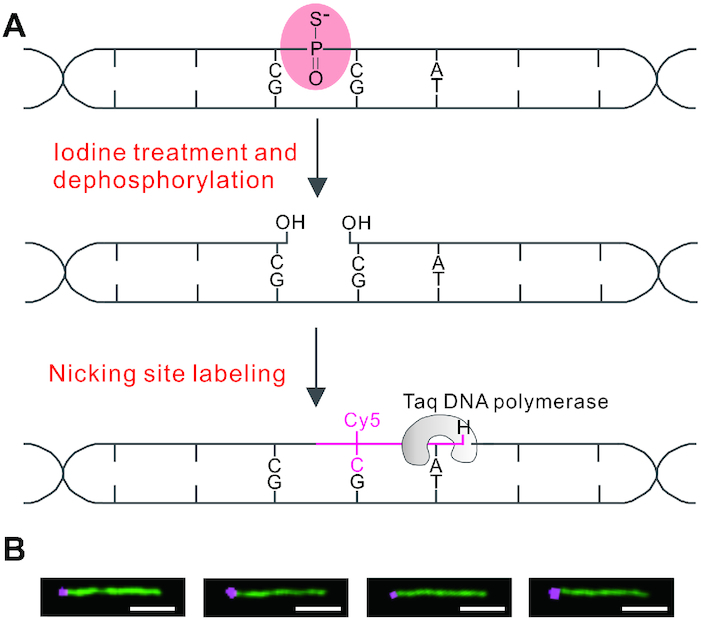
Scheme for selective fluorescent labeling of single-stranded PT modifications. (**A**) Upon iodine-induced strand cleavage and subsequent dephosphorylation with shrimp alkaline phosphatase, the single-stranded PT at 5′-C_PS_CA-3′ is converted to a DNA nick with a free 3′-OH, which is filled by Taq DNA polymerase-mediated sequential incorporation of 5-propargylamino-dCTP-Cy5 and dideoxyATP. (**B**) Selected images of the 15-kb-C_PS_CA molecules labeled with Cy5 (shown in violet) present close to the left end. The DNA backbone is stained with YOYO-1 (green); scale bar: 2.5 μm.

Iodine-induced cleavage at PT provides an opportunity to specifically convert single-stranded PTs to DNA nicks, which can be further filled with fluorescently labeled nucleotides by DNA polymerase extension using the opposite strand as a template. This approach is predicted to create a fluorescent readout along the DNA contour resembling beads on a string. The experimental concept is schematically depicted in Figure 1A. We first set out to validate this method with a proof-of-principle experiment by direct imaging of the chemically synthesized 5′-C_PS_CA-3′ located at one end of a 15-kb PCR fragment. After iodine treatment and dephosphorylation, the PT linkage was converted to a nick, leaving behind a 3′-OH end and rendering it available for fluorescent tagging by Taq DNA polymerase-mediated incorporation of 5-propargylamino-dCTP-Cy5 (Figure 1A). The DNA backbone was stained with the intercalating dye YOYO-1, stretched on chemically modified glass surfaces by molecular combing, and imaged with confocal microscopy. Figure 1B shows typical Cy5 dye-labeled 15-kb-C_PS_CA DNA molecules, confirming the labeling specificity of PT modification.

**Figure 2.**
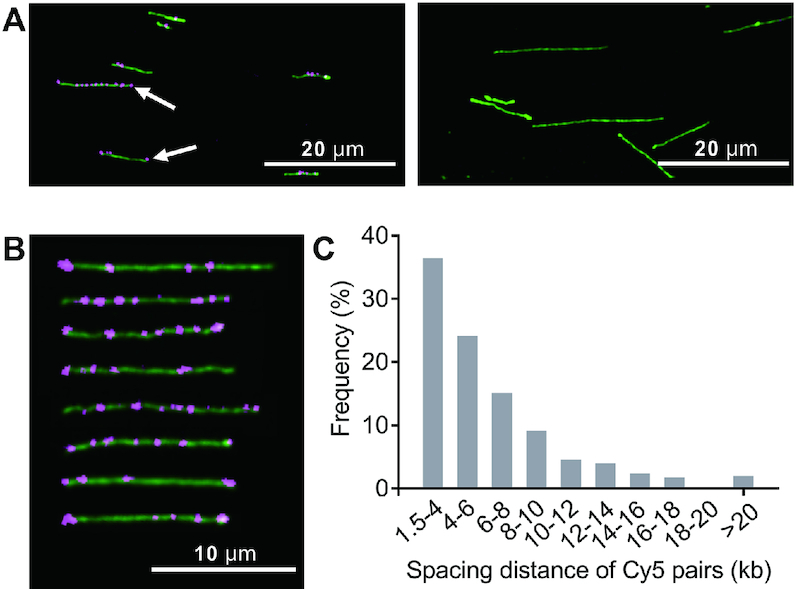
Optical detection of single-stranded PTs across the *E. coli* 3234/A genome. (**A**) Typical images of genomic DNA prior to (right panel) and after (left panel) iodine treatment and Cy5 labeling. White arrows indicate Cy5 labels located at DNA extremities. (**B**) Cropped images of *E. coli* 3234/A genomic DNA fragments labeled with Cy5. (**C**) Histogram showing the frequency of 502 measured distances between Cy5 pairs in genomic DNA of *E. coli* 3234/A.

We next tested our ability to optically detect PT modifications in genomic DNA. Prior to iodine cleavage, DNA isolated from *sspBCDE*-containing *E. coli* 3234/A was treated with DNA polymerase I and dideoxyNTPs (ddNTPs) to block pre-existing nicking sites as described previously (14). This step greatly decreased the labeling of pre-existing random nicks and ensured that the fluorescent signals are truly indicative of PT sites rather than leftover pre-existing nicks (Supplementary Figure S1). As shown in Figure 2A and B, the optical detection immediately revealed individual fluorescent spots along the genomic fragments, enabling investigation of PT modifications along single DNA molecules. Due to the lack of markers to indicate DNA orientation, spacing lengths between two neighboring Cy5 spots were measured and converted to kilobase pair (kb) values on the basis of the measured average length for 48.5-kb λ phage DNA, stretched to 94.2% of its B-DNA contour length (0.34 nm/bp) in our assays (Supplementary Figure S2). There are 194 797 5′-CCA-3′ sites across the genome of *E. coli* 3234/A (GenBank number: GCA_001637635.1), with a spacing length of 3–388 bp. However, a histogram of 502 measured distances between Cy5 pairs from several fields of view revealed that the distances were predominantly 1.5–4 kb (36.5%) and 4–10 kb (48.4%), whereas lengths >20 kb accounted for 2% (Figure 2C). This highlighted the fact that PT distribution is nonhomogenous along the *E. coli* 3234/A genome and that only a fraction of genome-wide 5′-CCA-3′ motifs were PT protected even in the presence of the restriction component SspE. Notably, we also observed apparent shortening of the DNA contour length upon iodine treatment, which most likely resulted from DNA double-strand breaks when two 5′-C_PS_CA-3′-derived nicks occurred too close to each other on opposite strands. This was supported by the observation that a number of fluorescent spots were located on the extremities of DNA fragments (Figure 2A).

### Molecule-to-molecule PT heterogeneity

**Figure 3.**
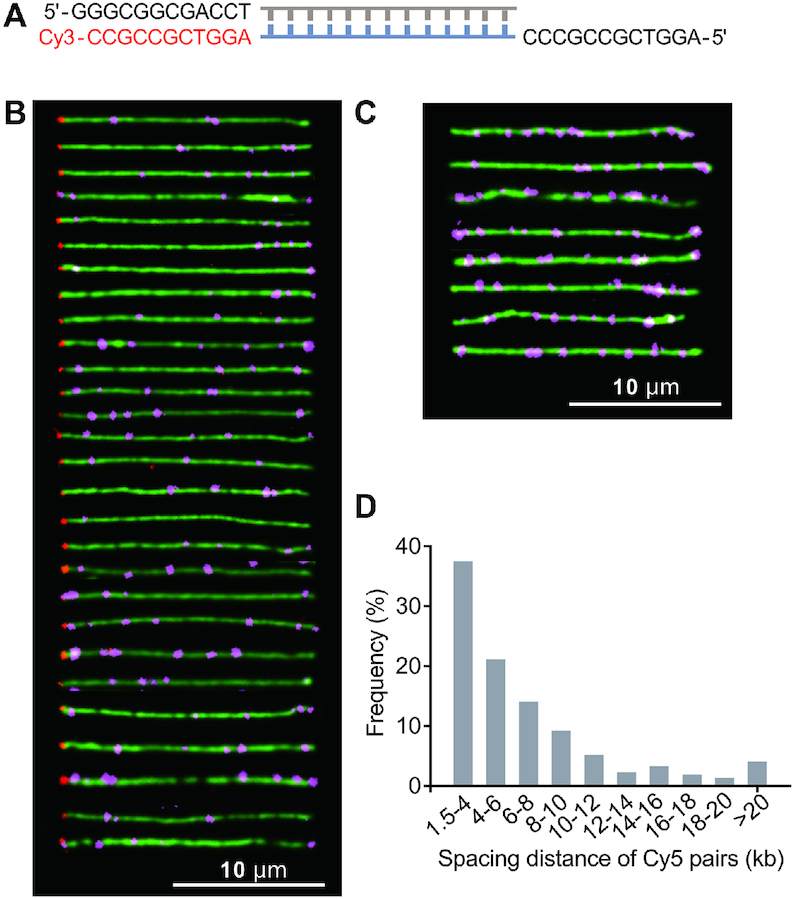
Optical mapping of single-stranded PT modifications along individual combed λ DNA molecules. (**A**) Orientation of the λ genome by ligating a Cy3-labeled oligonucleotide complementary to the 5′ overhang on the left end. (**B**) Cropped images of individual full-length λ genomes labeled with Cy5 at PT-modified 5′-C_PS_CA-3′ sites and with Cy3 (shown in red) at the left end. (**C**) Examples of λ DNA molecules decorated with Cy5 but lacking the Cy3 end label. (**D**) Histogram showing the frequency of 520 measured spacing distances between two neighboring Cy5 dots along λ genomes.

The optical imaging of PT in genomic DNA raised a question of fundamental importance: how do PT-modifying SspABCD proteins determine PT patterns to keep the restriction counterpart SspE in check in the face of such a state of partial PT modification? Here, we employed λ phage as a model to profile the PT distribution at the single-genome level. Enterobacteria phage λ is a well-characterized virus that infects *E. coli*. It contains a 48.5-kb double-stranded, linear genome with 12-base complementary single-stranded DNA segments at both 5′ ends (Figure 3A). When λ phage was passaged through an *E. coli* DSM 3925 (pWHU3639) strain expressing SspBCD, full protection against SspE defense was obtained, but this protection was subsequently abolished after the propagation of such phages on *ssp*-lacking *E. coli* DSM 3925, confirming that PT modification had occurred on λ DNA in the former scenario (Supplementary Figure S3). This allowed us to determine PT patterns that enable λ phage to bypass the restriction of the SspE barrier.

To construct the map of PT modifications, we established the orientation of λ DNA molecules by ligating a Cy3-labeled complementary oligonucleotide (5′-AGGTCGCCGCC-Cy3-3′) to the 5′ overhang on the left end according to Yardimci *et al.* (15) (Figure 3A). To understand PT occurrence along individual λ genomes, DNA molecules that fulfill either of the following two criteria were selected for analysis. In the first group, the full-length λ DNA was simultaneously decorated with two dyes, namely, Cy3 and Cy5, to ensure accurate PT profiling (Figure 3B). In the second group, the λ DNA fragments had to have at least two resolvable Cy5 moieties so that the relative distances between dyes could be determined (Figure 3C). The fluorescence images of a collection of λ genomes were clearly displayed in Figure 3B, revealing the numbers of Cy5 spots, their positions relative to the Cy3-labeled DNA extremity, and the spacing length between two neighboring Cy5 dots. Remarkably, the PT-modified λ genomes that had been recognized as self-DNA by the host Ssp defense system displayed significant molecule-to-molecule PT heterogeneity, excluding the possibility that SspE recognizes a specific PT pattern for self–non-self discrimination. This also explained why PT modification is partial at a given site in a population of DNA molecules, as observed by ICDS and SMRT sequencing (11,12). In total, one to twelve resolvable Cy5 moieties per λ genome were visualized, and the number varied from genome to genome. However, more PT modifications per λ genome should be expected because the uneven fluorescence intensity of the Cy5 spots indicated that a single fluorescent spot may contain more than one PT site due to the diffraction-limited resolution of optical imaging. This limited us to determine the precise numbers of PT sites along individual DNA molecules. Instead, we measured the distances between two resolvable Cy5 labels and found that 37.5% out of 520 measured distances between Cy5 pairs were in the range 1.5–4 kb and 44.4% ranged from 4 to 10 kb, which was consistent with the observed distances in genomic DNA of *E. coli* 3234/A (Figure 3D).

### Site-specific covalent labeling of double-stranded PT modifications

**Figure 4.**
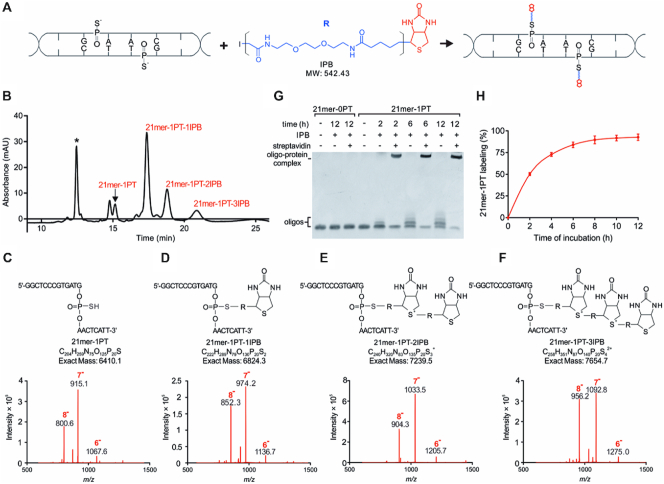
Selective chemical labeling of PT modifications. (**A**) Description of selective tagging of PT with IPB. (**B**) HPLC analysis of the reaction mixture containing 21mer-1PT and the biotinylated products, 21mer-1PT-1IPB, 21mer-1PT-2IPB and 21mer-1PT-3IPB. The peak, marked *, is a 20mer oligonucleotide (5′-GGAGCTGAGTGATCGCGTCA-3′) used as an internal standard to determine the labeling efficiency in (H). (**C–F**) ESI-MS (*m/z*) spectra of 21mer-1PT, 21mer-1PT-1IPB, 21mer-1PT-2IPB and 21mer-1PT-3IPB. All the spectra were recorded on a Thermo LCQ Deca XP ion trap mass spectrometer in negative ion mode. (**G**) The PAGE image showing the chemical labeling of 21mer-0PT and 21mer-1PT with IPB and subsequent conjugation with streptavidin. At indicated time points, the reaction mixture was ultrafiltered using centrifugal devices with Omega Membrane 1K (Pall) three times followed by incubation with 1.5 μM streptavidin for 1 h at 37°C. The resulting mixtures were loaded onto a 17% polyacrylamide gel and electrophoresed at 130 V in 0.5 × TBE buffer at 4°C and visualized by staining with GelRed. The 21mer-0PT sequence is the same as that of 21mer-1PT but lacks the PT modification. (**H**) Due to the diversity of products, labeling efficiency was reflected as the percentage of the decrease in substrate peak area according to the time of reaction. The values are the means ± standard deviations, *n* = 3.

Iodine treatment results in severe fragmentation of DNA harboring bistranded PT modifications (11), which is therefore not suitable for optical mapping of double-stranded PTs. The nucleophilic property of PT sulfur enables a number of chemical tagging strategies. It is noteworthy that the internucleotidic PT diester is less nucleophilic than the 5′- or 3′-terminal PT monoester, and previous labeling reactions were mainly carried out in organic buffer systems (16–18). Here, two important considerations have guided our approach: (i) the covalent labeling reaction should occur in a PT-specific and highly efficient manner, and (ii) the reaction should be conducted under very mild, nondenaturing conditions to avoid DNA conformational changes or damage. Here, the bifunctional molecule iodoacetyl-PEG2-biotin (IPB) was applied in PT-specific covalent labeling. IPB has three elements: an iodoacetyl group on one end that reacts with sulfhydryl groups by nucleophilic substitution, generating a thioether bond; a hydrophilic polyethylene glycol (PEG) spacer arm that makes the molecule water soluble; and a biotin group on the other end that allows subsequent attachment to fluorophores. A schematic illustration of this strategy is shown in Figure 4A.

After confirming that the covalent labeling of PT sites with IPB led to no DNA breakage (Supplementary Figure S4), we aimed to assess the labeling efficiency by selective tagging of a 5′-G_PS_AAC-3′ site in a 21-mer oligonucleotide (21mer-1PT) with IPB in phosphate buffer (10 mM, pH 7.0) at 50°C in the dark. At appropriate time intervals, aliquots were removed, desalted by ultrafiltration and subjected to analysis by high-performance liquid chromatography (HPLC). Along with the consumption of the 21mer-1PT substrate, a new peak gradually appeared on the chromatogram (Figure 4B). The electrospray ionization mass spectrometry (ESI-MS) analysis of this peak revealed ions at *m/z* 852.3, *m/z* 974.2 and *m/z* 1136.7, which correspond to [M-8H]^8−^, [M-7H]^7−^, and [M-6H]^6−^ ions of the iodoacetyl-PEG2-biotinylated PT-containing oligonucleotide, denoted 21mer-1PT-1IPB, respectively (Figure 4C and D). Interestingly, two minor peaks were observed that eluted later and yielded a series of multiply charged ions, which were assigned to 21mer-1PT covalently labeled with two and three IPB molecules, yielding 21mer-1PT-2IPB and 21mer-1PT-3IPB, respectively (Figure 4E and F). The chemical conjugation was further supported using matrix-assisted laser desorption/ionization-time of flight mass spectrometry (MALDI-TOF MS) analysis (Supplementary Figure S5). We reasoned that this could be attributed to the intermolecular self-alkylation of IPB between the dialkyl sulfide and iodoalkyl groups, similar to that reported in the case of isotope-coded affinity tag (ICAT) reagents (19). Indeed, polyacrylamide gel electrophoresis (PAGE) revealed more biotinylated derivatives of 21mer-1PT with increasing molecular weights resulting from the successive introduction of IPB moieties. Nevertheless, all the resultant conjugates mediated by IPB and self-alkylated derivatives were PT-specific and bound tightly to the receptor streptavidin (Figure 4G). In contrast, no band shift was detected when 21mer-0PT, with the same nucleotide sequence as 21mer-1PT but lacking the PT linkage, was subjected to the same labeling reaction, confirming that covalent labeling with IPB occurs specifically with the sulfhydryl group of the PT linkage (Figure 4G). With increasing reaction time, the peak absorbance of the starting 21mer-1PT substrate decreased and was barely observed after 12 h incubation, suggesting that the chemical labeling was largely complete. The coupling yield of 21mer-1PT was ∼92% at 12 h, as judged by HPLC analysis (Figure 4H).

### Optical detection of double-stranded PT modifications

**Figure 5.**
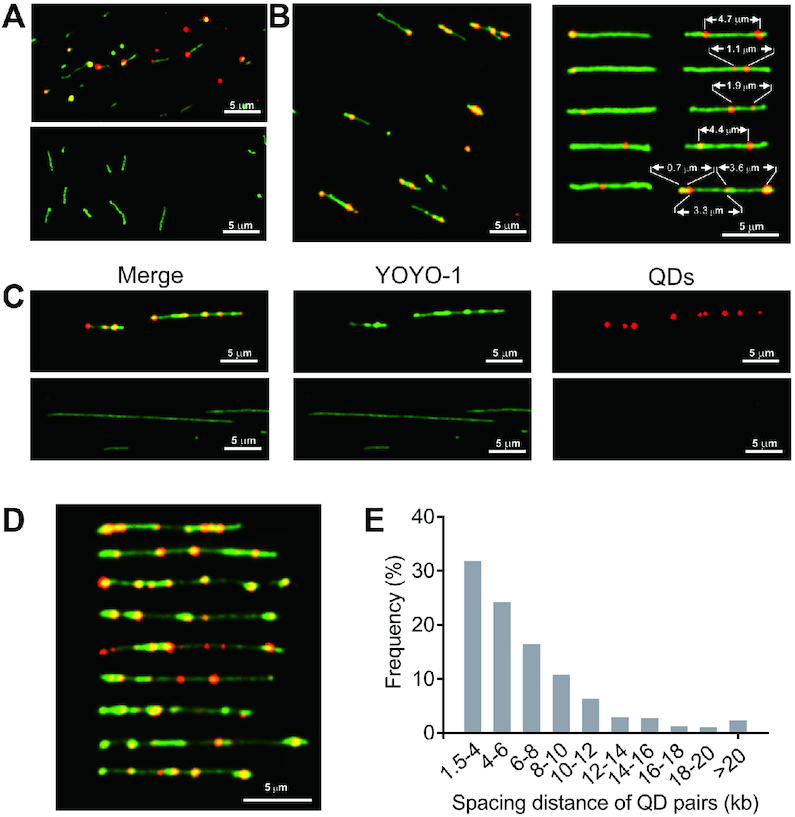
Optical detection of PT-specific tagging by quantum dots. Single-molecule images of flow-stretched, YOYO-1-stained 15-kb PCR products (**A**), linearized pWHU3930 plasmid DNA (**B**) and genomic fragments of *S. enterica* serovar Cerro 87 (**C** and **D**) with QDs (red). Overlapping red and green signals are shown in yellow. (A) QD signals located at the end of 15-kb-G_PS_AAC DNA molecules (upper panel), consistent with the position of 5′-G_PS_AAC-3′ sites. No QD signals were observed along the backbone of PT-lacking 15-kb-GAAC molecules (lower panel). (**B**) Images of NdeI-linearized pWHU3930 plasmid DNA labeled with QDs (left panel). The optical patterns of PT in selected plasmid DNA (right panel). (**C**) Optical detection of PTs across the *S. enterica* serovar Cerro 87 genome by conjugation with IPB and subsequent QD attachment (upper panel). DNA of Cerro 87 prior to QD attachment (lower panel). (**D**) Cropped images of Cerro 87 genomic DNA fragments labeled with QDs. (**E**) Histogram showing the frequency of 474 measured spacing distances between two QDs pairs along Cerro 87 genomic DNA fragments.

The selective biotinylation of PT modifications provided a strategy to light up PTs by using streptavidin-fluorophore conjugates, for example, streptavidin-coated fluorescent QDs. We demonstrated the utility of this approach by direct visualization of a PT-modified 5′-G_PS_AAC-3′ motif located at one end of an artificial 15-kb DNA fragment (15-kb-G_PS_AAC). Fluorescent spots indicating individual 5′-G_PS_AAC-3′ sites were clearly visible in the YOYO-1-stained DNA fragments (Figure 5A). In contrast, no QDs were detected in 15-kb-GAAC, with the same DNA sequence as 15-kb-G_PS_AAC but lacking the PT modification, which confirmed the PT-selective labeling with QDs (Figure 5A). Encouraged by the results, we then exploited this approach to optically map double-stranded PT modifications in natural DNA. The resulting dual-color fluorescence images clearly showed fluorescent QD spots on 19.9-kb pWHU3930 plasmid DNA molecules (Figure 5B, left panel; Supplementary Table S1). The PT modification sites in pWHU3930 were collapsed to between one to five resolvable QD sites, and some QD signals were apparently much stronger than others, suggesting that some 5′-G_PS_AAC-3′/5′-G_PS_TTC-3′ sites were closely clustered (Figure 5B, right panel). Nevertheless, the position of QDs and relative distances between two resolvable QD labels varied markedly from molecule to molecule, indicating PT heterogeneity similar to that observed in single-stranded PT modifications (Figure 5B, right panel).

We next validated the utility of such an approach to probe DNA PT modifications in genomic DNA. Figure 5C and D show color overlay images of genomic DNA of *S. enterica* serovar Cerro 87 decorated with a string of QDs. Interestingly, 31.9% and 51.5% of the 474 measured distances between QD signals were predominantly in the ranges of 1.5–4 kb and 4–10 kb, respectively, which resembled the distances observed in single-stranded PT systems (Figure 5E). Collectively, these data indicated that regardless of the resultant double- and single-stranded PT modifications, DndABCDE and SspABCD systems, respectively, likely share a similar mechanism to select a small fraction of sites from all available consensus sequences across individual DNA molecules for sulfur incorporation. The diversity of PT patterns in a population of DNA molecules prompted us to hypothesize that in addition to the PT status of consensus sequences, more elements should be considered in the self–non-self discrimination and restriction functions of DndFGH and SspE, such as the spacing length between two adjacent PT sites and PT density in a single DNA molecule.

## DISCUSSION

The study presented here was motivated by our observation that DNA PT modification occurred only in a proportion of genome-wide modifiable consensus sites despite the presence of restriction cognates (11). This feature differentiates the PT-based Dnd and Ssp systems from classic methylation-based epigenetic and R-M mechanisms. The current genomic PT mapping methods of ICDS and SMRT sequencing compile long-range sequences from the assembly of numerous short sequence reads and thereby report on the population-averaged distribution of PT modifications (11,14). The characterization of PT distribution along individual DNA molecules is therefore essential to further our understanding of PT physiology and may provide insights into the unusual behaviors of target selection in Dnd and Ssp systems. In this study, we developed two single-molecule approaches to directly visualize PT modifications; these approaches remove the usual ensemble average and provide access to information such as the PT status of long genomic regions and molecule-to-molecule PT variation. The two approaches are easy to implement and consist of several key steps: iodine-mediated chemical cleavage or covalent labeling of PT to enable the site-specific incorporation of fluorophores; extending DNA molecules to linear form on chemically modified glass surfaces; and optical detection of fluorescent dye molecules along the DNA backbone. In addition to DNA damage sites (20), 5-methylcytosine (21) and 5-hydroxymethylcytosine (22), we added PT to the repertoire of information available for optical mapping and extended the utility of optical mapping to assess epigenetic markers.

Optical mapping immediately revealed that PTs do not occur in a specific pattern in either Dnd or Ssp systems, with varying PT positions among DNA molecules. Although DndABCDE and SspABCD involve different sets of enzymes that confer PT modifications, there is a high probability that they share a common mechanism to choose targets from the large number of modifiable consensus sites in individual DNA molecules, leading to PT heterogeneity. Our previous studies have shown that deletion of *dndB*, the transcriptional repressor of *dndCDE* and itself, in *S. enterica* serovar Cerro 87 caused a 2-fold increase in the total PT frequency (9). This result suggested that the vacant 5′-GAAC-3′/5′-GTTC-3′ sites are still amenable to PT modification with increasing expression of DndACDE proteins. In combination with the optical mapping of PTs, this result raises the possibility that the ‘density’ of PT modifications in DNA is regulated by the cellular DndACDE and SspABCD protein abundance (12). Based on the observation that PT modification is capable of influencing gene transcription by RNA polymerase *in vitro*, cell-to-cell PT heterogeneity is predicted to lead to heterologous gene transcriptional profiles, which might provide a fitness advantage to hosts in changing environments (6,7).

The fitness advantage resulting from PT heterogeneity is a reasonable speculation regarding the reason for the solitary DNA PT modification systems. However, what keeps the restriction components, that is, DndFGH and SspE, in check in the presence of the state of incomplete PT modification? Research into the enzymatic activity of DndFGH is currently limited due to the complex composition, whereas SspE is better understood (2). SspE inhibits phage replication by virtue of its DNA-nicking nuclease activity, introducing massive nicks into phage genomes (2). Interestingly, SspE possesses additional NTPase activity that is stimulated specifically by DNA fragments with a 5′-C_PS_CA-3′ motif (2). NTPase activity is essential to the antiphage activity of SspE, rendering SspABCD-SspE a PT-sensing defense barrier. This PT-stimulated NTPase activity is predicted to help with SspE translocation or movement along DNA molecules (2). Together with the observation that the distribution of PT is discrete but the spacing distances between two neighboring 5′-C_PS_CA-3′ sites in the *E. coli* 3234/A and λ genomes and two 5′-G_PS_AAC-3′/5-G_PS_TTC-3′ sites in the *S. enterica* serovar Cerro 87 genome are predominantly <10 kb, we propose that (i) DndFGH and SspE may employ a similar strategy to distinguish self from nonself DNA, and (ii) in addition to sequence-specific PT modifications, they likely require additional information to accomplish self-nonself discrimination and restriction, for example, PT density in a range of DNA fragments. We speculate that the spacing distance between PT modification sites is involved in the protection of self-DNA against the restriction of DndFGH and SspE. If it is too far between the two PTs in the genome, it may not be able to provide sufficient protection from the cleavage by restriction components. Moreover, in parallel with the cell-to-cell PT heterogeneity, one could envision a scenario in which DndFGH or SspE does not exert restriction function consistently at a given site and, consequently, behaves differently in cells to avoid self-restriction. However, the unusual heterogeneity feature may hinder exploration of the interaction between PT-modified DNA substrates and DndFGH or SspE *in vitro*.

Iodine-mediated nick conversion at PT sites enables the optical mapping of PT modifications along single viral genomes as well as prokaryotic genomic DNA fragments, providing information on PT distribution and relative distance between two resolvable PT sites. In addition to the fluorescently labeled dCTP used in this study, diverse nucleotide derivatives can be used to indicate PTs, which potentially extends the detectability of PT by multiple techniques. For instance, Zatopek *et al.* reported a Rare Damage and Repair sequencing (RADAR-seq) method in which DNA lesions are replaced with a patch of methylated nucleotides allowing the direct detection by SMRT sequencing (23). In terms of selective chemical labeling of PT by fluorescent dyes, this approach is technically applicable to optical detection of both single- and double-stranded PT modifications. It is worth mentioning that when DNA molecules are stretched by the drag force of the receding meniscus, the force scales with the cross-sectional area of the objects experiencing it, that is, DNA or QD. One can therefore imagine that the extension force is increased due to the larger diameter of QD (24,25). We noticed that DNA decorated with a cluster of QDs is more vulnerable to molecular combing. This problem could be addressed by adopting alternative DNA-stretching strategies, that is, nanochannel technology (26). While the two optical approaches offer the genomic context of PT modifications, they lack the base-level resolution of sequencing. Of note, we also tried to subject IPB-labeled PT DNA for SMRT sequencing, aiming to amplify the detection signal at PT sites or read potential terminal points. However, no sufficient data were generated, which is presumably attributed to the hindrance of the bulky IPB moieties to the polymerase-mediated synthesis process in SMRT sequencing. Similarly, the bulky group of IPB at PT sites might have hindered the passage of DNA molecules through the nanopores during nanopore sequencing, resulting in a lack of data (27). Nevertheless, PT-labeling-based sequencing requires further exploration for single-molecule base-level resolution of PT mapping, which will be the focus of our future work.

In summary, we have developed two optical mapping approaches to create PT-derived fluorescent read-out along individual stretched DNA molecules, allowing the exploration of the genomic PT variation inaccessible by previous techniques and providing novel insights into the unusual target selection mechanisms of both Dnd and Ssp systems.

## Supplementary Material

gkab169_Supplemental_File
