# Supplementary material for: Single-molecule optical mapping of the distribution of DNA phosphorothioate epigenetics
Source: Nucleic Acids Res. 2021 Mar 25;49(7):3672–80. doi: 10.1093/nar/gkab169 (PMC8053081; doi:10.1093/nar/gkab169)
Supplement: gkab169_Supplemental_File [file gkab169_supplemental_file.pdf]

Supplementary Information

Single-molecule optical mapping of the distribution of DNA phosphorothioate epigenetics

Supplementary Table S1. Strains, plasmids and phages used in this study

Strain, plasmids and phages	Characteristics	Source or reference
**strains**		
E. coli DH10B	F- *mcrA* Δ(*mrr-hsdRMS-mcrBC*) φ80*dlacZ*ΔM15 *ΔlacX74 recA1* endA1 araD139 Δ (ara, leu)7697 *galU galK rpsL nupG λ*-	Invitrogen
E. coli 3234/A	d(C_{PS}C), GenBank: LCVH01000033	Gift from Prof. Ulrich Dobrindt
E. coli DSM 3925	F^- *ton*A21 *thi*-1 *thr*-1 *leu*B6 *lac*Y1 *gln*V44 *rfb*C1 *fhu*A1 λ^-	CCTCC
S. enterica serovar Cerro 87	d(G_{PS}A) and d(G_{PS}T), GenBank: CP008925.1	(1)
**plasmids**		
pBluescript II SK(+)	Cloning vector, 3 kb, *bla*	(2)
pWHU3930	19.9 kb, pBluescript II SK(+) derivative expressing the *dndBCDE-dndFGH* cassette from *S. enterica* serovar Cerro 87	This work
pWHU3639	8.1 kb, pBluescript II SK(+) derivative with *sspBCD* from *E. coli* 3234/A	This work
pWHU3640	10.8 kb, pBluescript II SK(+) derivative with *sspBCDE* from *E. coli* 3234/A	(3)
**phage**		
λ	48.5 kb double-stranded DNA, *Siphoviridae*	CCTCC

Supplementary Table S2. Primer and probe sequences used in this study

Primers	Sequences
15-kb-U	TTACTAATGAATTCAGGACAGACAGTG
15-kb-C_{PS}CA-D	AACCTCTCTGTTTACTGATAAGTTC$_{PS}$CA
15-kb-D	AACCTCTCTGTTTACTGATAAGTTCCA
15-kb-F	CGCCGCCTCTTTTCATCTCACTACCAC
15-kb-G_{PS}AAC-R	CCTGCATCCTG$_{PS}$AACCCATTGACCTCC
15-kb-R	CCTGCATCCTGAACCCATTGACCTCC
Cy3-labeled-probe	AGGTCGCCGCC-Cy3
21mer-1PT	GGCTCCCGTGATG$_{PS}$AACTCATT
21mer-0PT	GGCTCCCGTGATGAACTCATT
20mer	GGAGCTGAGTGATCGCGTCA

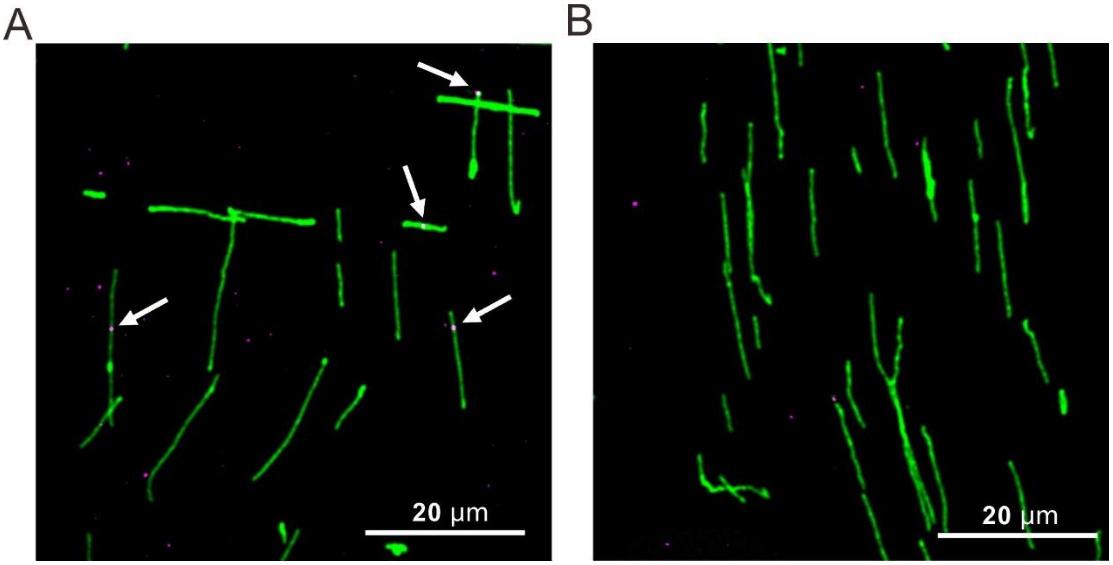

Supplementary Figure S1. Pre-existing random nicks of *E. coli* 3234/A genomic DNA were blocked by ddNTPs. Typical images of genomic DNA prior to (**A**) and after (**B**) blocking by ddNTPs and Cy5 labeling. White arrows indicate Cy5 labels located at pre-existing random nicks.

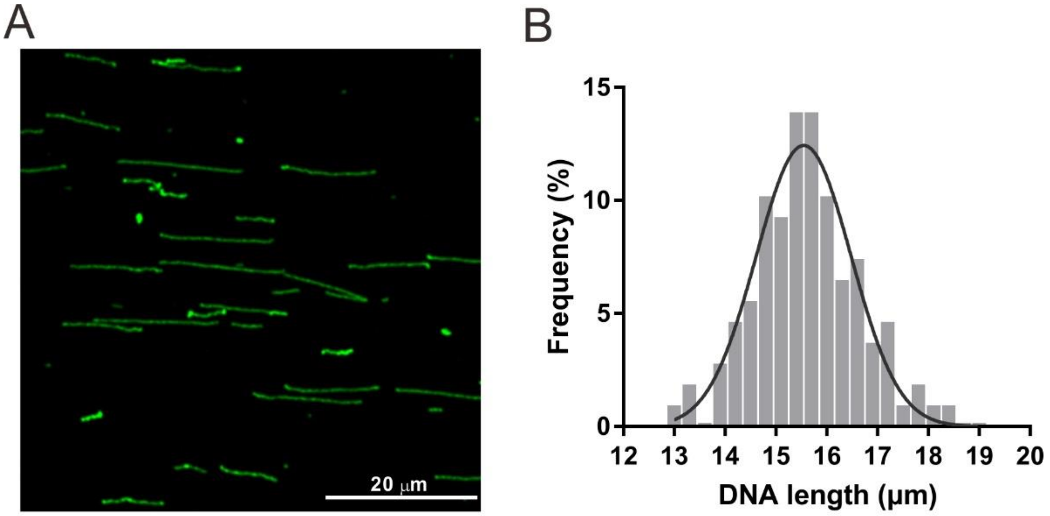

Supplementary Figure S2. Extension uniformity of λ DNA. (**A**) Optical image of full-length λ DNA stretched on silanized coverslips. (**B**) Frequency distribution histogram constructed from 108 full-length λ DNA molecules. The center of the Gaussian fit to this histogram is 15.5 ± 0.9 μm, corresponding to 94.2% of its B-DNA contour length.

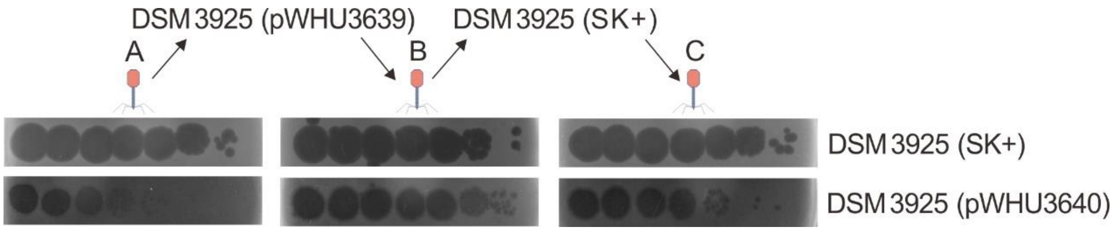

Supplementary Figure S3. DNA PT modifications enabled phage λ to overcome the Ssp defense. (**A**) Lawn plates of *E. coli* DSM 3925 containing empty vector pBluescript II SK(+) or pWHU3640 were spotted with λ phage. Plasmid pWHU3640 is the derivative of pBluescript II SK(+), expressing the SspBCDE barrier from *E. coli* 3234/A. Phages propagated on DSM 3925(pWHU3639) (**B**), expressing the PT-modifying components SspBCD, and DSM 3925(SK+) (**C**) were collected to reinfect DSM 3925(SK+) and DSM 3925(pWHU3640) cells, respectively, at 37 °C. Phage λ propagated on DSM 3925(pWHU3639) was no longer sensitive to the Ssp defense, suggesting that DNA PT modifications had occurred in phage genomes.

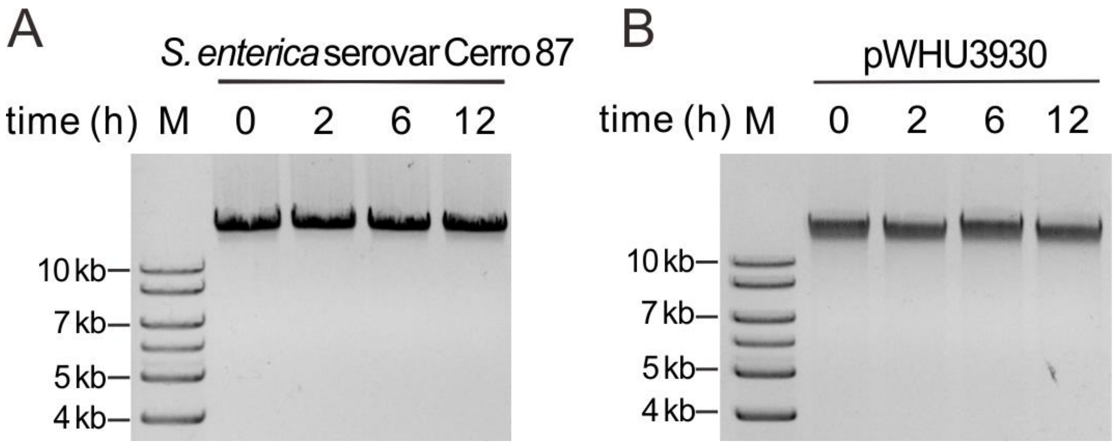

Supplementary Figure S4. Assessment of DNA integrity by gel electrophoresis. The integrity of genomic DNA of *S. enterica* serovar Cerro 87 (**A**) and plasmid pWHU3930 DNA (**B**) was assessed after the labeling reaction with IPB for the indicated time periods.

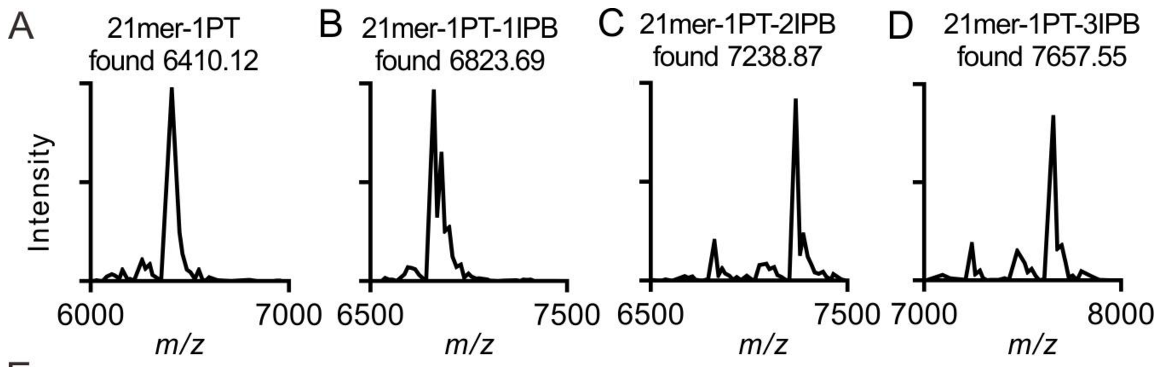

E

Reactant or product	Chemical Formula	m/z [M-H]$^-$	
		Calcd	Found
21mer-1PT	$C_{204}H_{259}N_{75}O_{125}P_{20}S$	6409.06	6410.12
21mer-1PT-1IPB	$C_{222}H_{289}N_{79}O_{130}P_{20}S_2$	6823.26	6823.69
21mer-1PT-2IPB	$C_{240}H_{320}N_{83}O_{135}P_{20}S_3^{+}$	7237.45	7238.87
21mer-1PT-3IPB	$C_{258}H_{351}N_{87}O_{140}P_{20}S_4^{2+}$	7651.64	7657.55

Supplementary Figure S5. The MALDI-TOF MS (negative ion) spectra of 21mer-1PT (**A**), 21mer-1PT-1IPB (**B**), 21mer-1PT-2IPB (**C**) and 21mer-1PT-3IPB (**D**). (**E**) The calculated and detected *m/z* of each molecule are listed.

References

1. Xu, T., Yao, F., Zhou, X., Deng, Z. and You, D. (2010) A novel host-specific restriction system associated with DNA backbone S-modification in Salmonella. *Nucleic Acids Res*, **38**, 7133-7141.
2. Alting-Mees, M.A. and Short, J.M. (1989) pBluescript II: gene mapping vectors. *Nucleic Acids Res*, **17**, 9494.
3. Xiong, X., Wu, G., Wei, Y., Liu, L., Zhang, Y., Su, R., Jiang, X., Li, M., Gao, H., Tian, X. *et al.* (2020) SspABCD-SspE is a phosphorothioation-sensing bacterial defense system with broad antiphage activities. *Nat Microbiol*, **5**, 917–928.
